# Critical assessment of knowledge-based models for craniospinal irradiation of paediatric patients

**DOI:** 10.1016/j.phro.2025.100703

**Published:** 2025-01-20

**Authors:** Paolo Caricato, Francesca Cavagnetto, Silvia Meroni, Salvina Barra, Laura Brambilla, Enrica Bovo, Samuele Cavinato, Alessio Cirone, Flavio Giannelli, Marta Paiusco, Emilia Pecori, Emanuele Pignoli, Margherita Pollara, Giovanni Scarzello, Alessandro Scaggion

**Affiliations:** a Medical Physics Department, Veneto Institute of Oncology IOV - IRCCS, Padova, Italy; b Medical Physics Department, IRCCS Ospedale Policlinico San Martino, Genova, Italy; c Medical Physics Unit, Fondazione IRCCS Istituto Nazionale dei Tumori, Milano, Italy; d Radiation Oncology Department, IRCCS Ospedale Policlinico San Martino, Genova, Italy; e Postgraduate school of Medical Physics, Università degli studi di Milano, Milano, Italy; f Radiation Oncology Department, Veneto Institute of Oncology IOV - IRCCS, Padova, Italy; g Life Science Computational lab (LISCOMPlab), IRCCS Ospedale Policlinico San Martino, Genova, Italy; h Radiation Oncology Department, Fondazione IRCCS Istituto Nazionale dei Tumori, Milano, Italy

## Abstract

**Background and purpose:**

Knowledge-Based Planning (KBP) is increasingly used to standardize and optimize radiotherapy planning. This study aims to develop, refine, and compare multicentric KBP models for craniospinal irradiation (CSI) in pediatric patients.

**Materials and methods:**

A total of 113 CSI treatments from three Italian centers were collected, comprising Computed Tomography scans, target and organ contours, and treatment plans. Treatment techniques included Helical Tomotherapy (HT) and Volumetric Modulated Arc Therapy (VMAT). Three KBP models were developed: a full model (F-model) using data from 87 patients, a reduced model (R-model) based on a subset of the same sample, and a replanned model (RP-model) using KBP re-optimized plans. Models’ quality was evaluated using goodness-of-fit and goodness-of-prediction metrics, and their performance was assessed on a validation set of 26 patients through dose-volume histogram (DVH) comparisons, prediction bias, and variance analysis.

**Results:**

The F-model and R-model exhibited similar quality and predictive ability, reflecting the variability of the original dataset and resulting in broad prediction intervals in low to mid-dose ranges. The RP-model achieved the highest quality, with narrower prediction bands. The RP-model is preferable for standardizing planning across centers, while the F-model is better suited for quality assurance as it captures clinical variability.

**Conclusions:**

KBP models can effectively predict DVHs despite extreme geometric variability. However, models trained on highly variable datasets cannot simultaneously achieve high precision and accuracy. Comparing KBP models is essential for establishing benchmarks to meet specific clinical goals, particularly for complex pediatric CSI treatments.

## Highlights

• Knowledge-based models for pediatric Cranio Spinal Irradiation have been built on 113 patients coming from three Institutions.
• A knowledge-based model reproducing patients and practice variability allows the possibility to expedite RTQA programs.
• A properly tailored knowledge-based model can be used to foster treatment homogenisation in terms of OAR sparing.
• The predictive accuracy of KBP models can be achieved standardising upfront the clinical practices.

## 1 Introduction

Radiation therapy (RT) is vital for achieving local control in the treatment of paediatric tumours, but it poses unique challenges in this population. Limited pediatric RT cases reduce institutional expertise, leading to variations in plan quality and compliance with clinical trials [1], [2]. Craniospinal irradiation (CSI) is particularly complex due to the wide anatomical variability across age groups, the lack of robust data on organ-at-risk (OAR) dose tolerances, and the numerous trade-offs required to balance dose distributions. Variability in treatment planning is further compounded by differences in institutional practices and individual planner experience, making consistency difficult to achieve [3].

The advent of Knowledge-Based Planning (KBP) offers a promising solution by reducing variability due to planner expertise [4] and inter-center practice differences [5]. KBP has proven effective as a decision-support tool in centralized quality assurance (QA) programs, enabling consistent treatment plans in clinical trials [5], [6], [7], [8]. Collaboration among experienced centers can generate robust predictive models, reducing OAR dose variability and providing guidelines for achievable constraints [9], [10], [11], [12], [13], [14].

In this study, three Italian centers, collectively managing approximately 50 % of Italy's pediatric patients and participating as national reviewers in the SIOP trial for medulloblastoma [15] [NCT02066220], collaborated to develop, refine, and evaluate multicentric KBP models. The goal was to provide a shared benchmark for optimizing and standardizing CSI treatments in pediatric patients.

## 2 Materials and methods

**Fig. 1:**
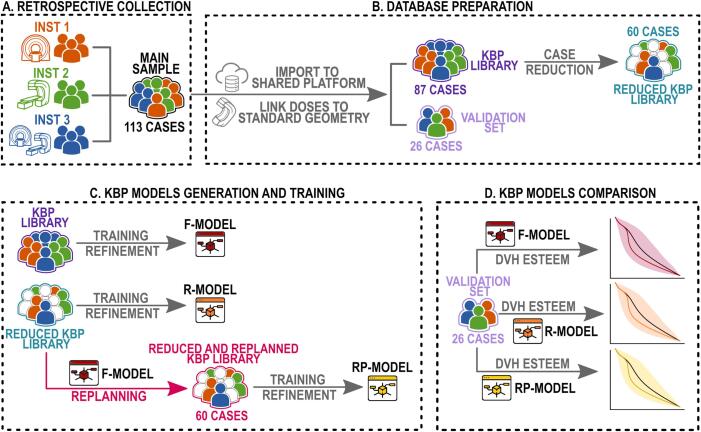
Visual representation of the study workflow.

Fig. 1 visually represents the workflow followed during the study, every section is explained separately in the following.

### 2.1 Retrospective collection

Patients treated with CSI for typical paediatric pathologies between 2016 and 2022 at three different Italian centres were included. A total of 113 cases were collected: 37 from Inst1 planned on Helical Tomotherapy (HT); 39 from Inst2 planned with Volumetric Modulated Arc Therapy (VMAT); and 37 from Inst3 19 planned with VMAT and 18 on HT (details in the Supplementary Material). To maximize sample size, adults were included, as size variability no longer correlates with age beyond 18 years [16].

The participating institutions have a history of collaboration and collectively serve as the Italian national QA committee for the PNET5 trial [15]. This study adhered to SIOP PNET5 and HRMB trial guidelines for standard and high-risk medulloblastoma, ensuring consistent practices across centers.

Computed Tomography (CT) scans with <3 mm slice thickness were acquired, positioning patients head-first supine with immobilization devices, including vacuum cushions, head and shoulder masks, and knee/feet fixation devices. The cranial clinical target volume (CTV) included the brain, cranial nerves, and meninges, while the spinal segment encompassed the spinal canal to the cerebrospinal fluid and ganglia, extending to the thecal sac based on co-registered MRI. A 5 mm isotropic expansion defined the planning target volume (PTV), although margins varied slightly by center and time. OARs such as eyes, lenses, thyroid, heart, lungs, and kidneys were mandatorily contoured, while others (e.g., parotid glands, breasts, and liver) were optionally delineated [17].

Treatment plans were optimized using institution-specific treatment planning systems (TPS): Precision (Accuray, USA) or Raystation (RaySearch, Sweden) for HT plans, and Eclipse (Varian, USA) for VMAT plans. VMAT plans used one isocenter for the brain and 1–2 for the spine, depending on the target length. To manage junctions, one centre applied a moving junction technique [15], while another ensured robustness against isocenter shifts by overlapping fields >5 cm with auto-feathering optimization in Eclipse [18]. Either an avoidance sector or beam no-entry through the patient’s arms was used. Photon energy was 6 MV or 6FFF MV, depending on delivery platforms. Further technical details are available in the Supplementary Material.

The clinical practices of the three institutions were compared in terms of ROIs volumes and relevant DVH metrics.

Ethical approval was obtained from all participating institutions (CESC IOV 2020/161, CER Liguria 255/2021 − DB id 11427, CESC INT N. 144/21).

### 2.2 Database preparation

All collected data were imported into the Eclipse AcademicHUB (Varian, USA) virtual platform, where subsequent analysis was conducted.

Historical dose distributions from clinically approved plans were associated with a standardized VMAT plan geometry, enabling the inclusion of HT dose distributions in RapidPlan [19]. This approach harmonized treatment practices across centers and ensured geometric consistency in the KBP model.

The VMAT plan geometry used one isocenter at the skull base with four full coplanar arcs. Two arcs, with collimator angles of 87° and 93°, covered the brain, while two with 357° and 3° targeted the upper PTV spine and part of the brain. Additional arcs were added to cover the remaining spine segments, incorporating one or two junctions based on target length. To maintain consistency, avoidance sectors were not applied. Details are provided in the Supplementary Material.

The dataset of 113 cases was divided into an 87-case KBP training library (40 HT, 46 VMAT) and a 26-case validation set (14 HT, 12 VMAT). Both samples had comparable age distributions and representation from the three centers. A reduced KBP library of 60 cases was also created by removing approximately one-third of the training set while maintaining similar age distributions and institutional proportions. This smaller dataset preserved anatomical variability while facilitating an evaluation of sample size effects on KBP model performance.

### 2.3 KBP models generation and training

The largest KBP library (87 cases) was used to train the full sample model (*F-model*). A restricted KBP model (*R-model*) was created using the reduced 60-cases library. Characteristics of both libraries are summarized in Supplementary Table 3. The same reduced dataset was used to generate replanned plans, leveraging DVH predictions from the *F-model*. Optimization included an initial automatic round followed by manual adjustments by an experienced planner. Standard plan geometry was maintained, using a single linac, 6 MV photons, and avoiding direct beam entrance through patients’ arms. The newly optimized plans formed the basis of a revised library, from which the *RP-model* was trained. This *RP-model* represents a hypothetical in-silico planner reflecting consistent planning habits standardized across the three participating centers.

The RP- and R-models, based on the same 60-case library, differed in their source data: the R-model used historical clinical plans, while the RP-model relied on KBP-optimized plans. This design preserved the inherent anatomical variability of the full dataset while allowing a direct evaluation of the impact of standardized replanning on model performance.

Training and data extraction followed recommendations from the Varian User’s Manual and relevant literature [6], [20], [21], [22]. Statistical outliers identified by RapidPlan tools were re-evaluated, with five cases undergoing re-optimization to improve plan quality.

All three KBP models are freely available at the Zenodo repository [23].

### 2.4 KBP models comparison

The quality of the three models was assessed using R^2^, χ^2^, and Mean Square Error (MSE). R^2^ quantifies variance explained by the model, χ^2^ evaluates goodness-of-fit, and MSE measures discrepancies between predicted and original DVHs, with lower MSE indicating better predictions [20].

The predictive performance of the models was evaluated on the validation set of 26 patients. Each model generated DVH predictions for OARs, which were compared to clinically approved historical DVHs using the method by Covele et al. [24]. Prediction accuracy was quantified by the mean systematic prediction error (prediction bias), calculated as the average point-by-point difference between predicted and historical DVHs across all validation cases. Statistical uncertainty was represented by the standard deviation (σ) of these differences. A prediction success rate can be also determined by comparing the clinical DVHs with patient-specific prediction errors from RapidPlan, calculating the proportion of dose points within the RapidPlan-generated prediction bands.

## 3 Results

### 3.1 Comparison of clinical practices

The substantial anatomical variability among patients, as shown in Supplementary Fig. 2, made it difficult to distinguish inter-center differences in contouring from those due to patient size. Both intra- and inter-center contour variability appeared comparable.

**Fig. 2:**
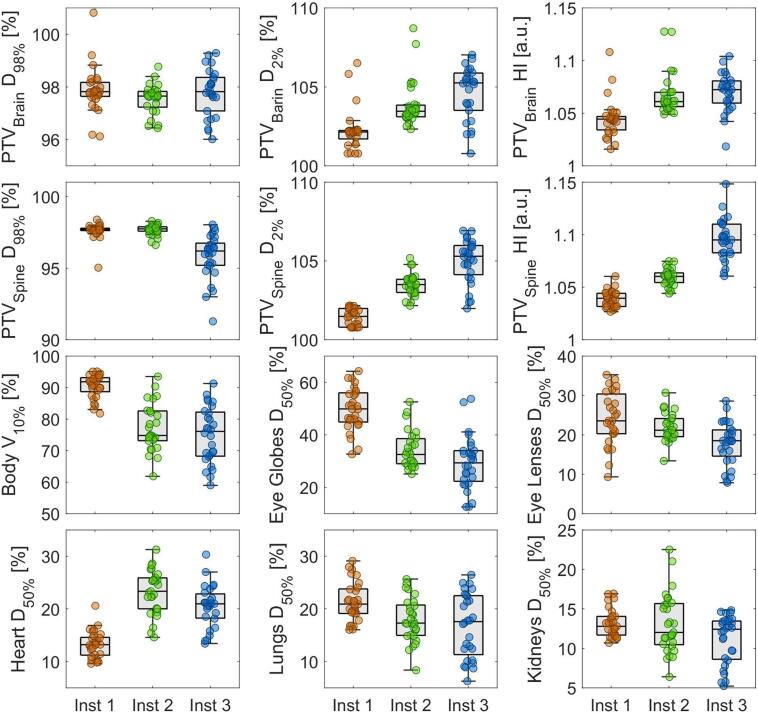
Box plots of relevant dosimetric quantities for PTVs and main OARs among the historical plans included in the whole dataset by the three institutions (113 treatment plans showed).

Fig. 2 highlights differences in clinical practices across the three centers. Inst3 exhibited the largest variability in target coverage and homogeneity (e.g., D_98%_, D_2%_, and Homogeneity Index), while Inst1 achieved the highest homogeneity. For OARs, different trade-offs were evident: Inst1 and Inst3 prioritized opposing compromises between doses to optical structures and PTV brain D_98%_, as well as between lung D_50%_ and heart D_50%_, with Inst2 consistently falling between these extremes.

Inst3 focused on sparing OARs, while Inst1 produced a higher integral dose. These differences were reduced during the replanning process, as shown in Supplementary Fig. 3.

**Fig. 3:**
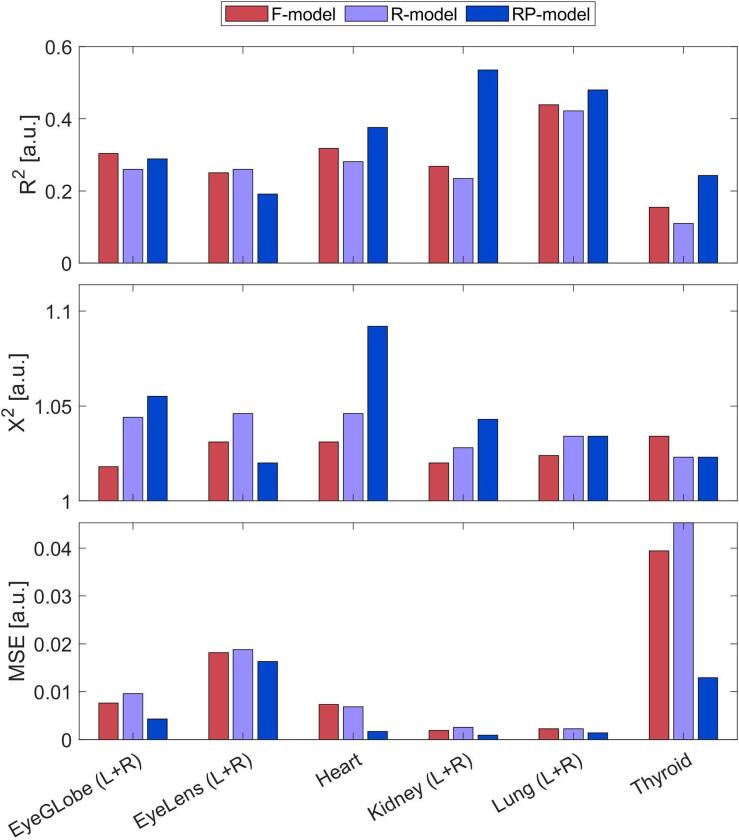
Comparison of models’ characteristics for the main OARs. From the top: Goodness of fit − R^2^ , Goodness of fit − X^2^ , Goodness of estimation − MSE.

### 3.2 Comparison of KBP models’ quality

Supplementary Table 1 summarizes the key characteristics of the three models' libraries, showing no significant differences in patient cohorts. Detailed statistical results are provided in Supplementary Table 2, while key metrics for relevant OARs are shown in Fig. 3.

The *F-model* demonstrated slightly better quality than the *R-model*, with higher R2, lower χ2, and lower MSE values. This indicates that reducing the library size by one-third had a minimal impact on the model’s ability to represent the sample. The *RP-model*, derived from replanned cases, achieved the highest R^2^ and lowest MSE, except for lenses. Its improved quality stemmed from the homogeneity of its training library, achieved through replanning guided by the *F-model* [4], [7], [25], [26]. These results highlight that library homogeneity, rather than size alone, has a greater influence on model quality.

### 3.3 Comparison of KBP models’ performance

**Fig. 4:**
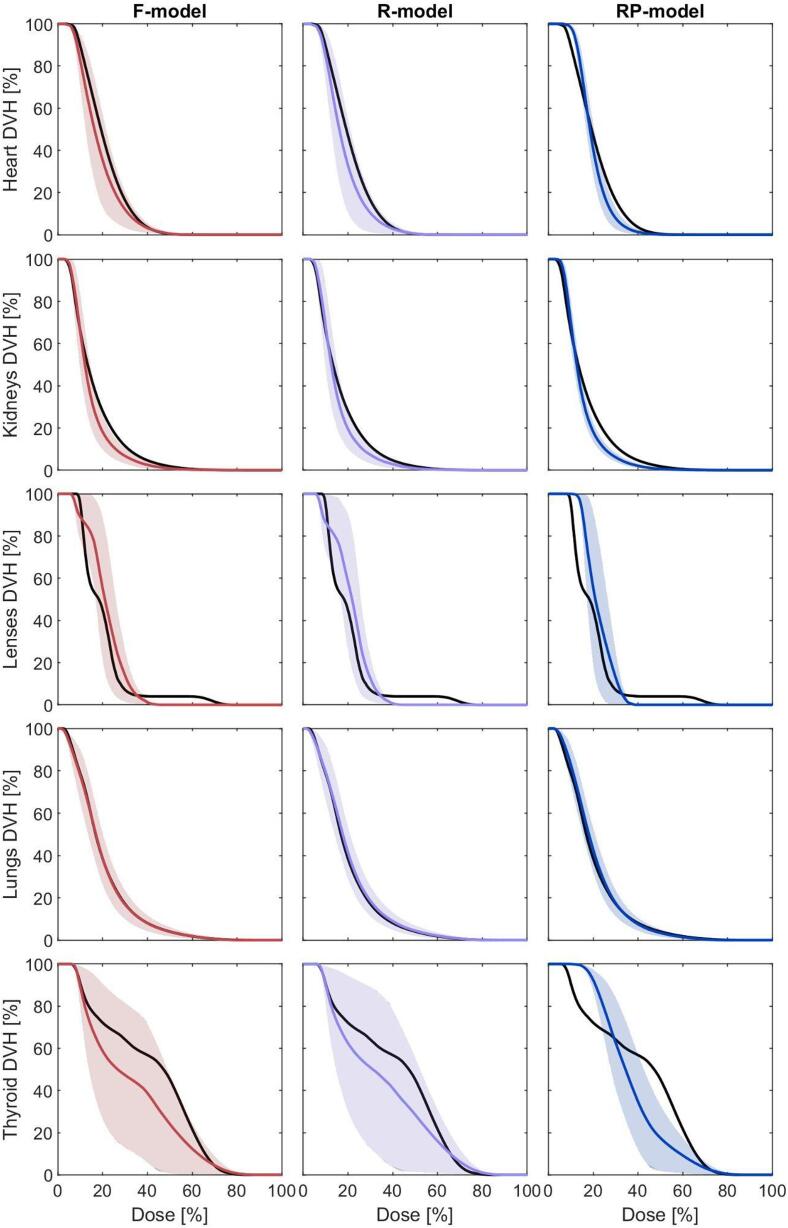
Models’ performance comparison on the validation set. The black line represents the mean historical DVH, the coloured line represents the mean RapidPlan predicted DVH, and the shaded area represents the mean RapidPlan prediction uncertainty.

Fig. 4 compares the performance of the three models on the validation set for main OARs. The *RP-model* exhibited narrower prediction bands, reflecting higher precision for most OARs, except the lenses, where its bands were comparable to the other models. Mean historical DVHs generally fell within the prediction bands of the *F-* and *R-models* but deviated from the narrower *RP-model* bands for many OARs.

**Fig. 5:**
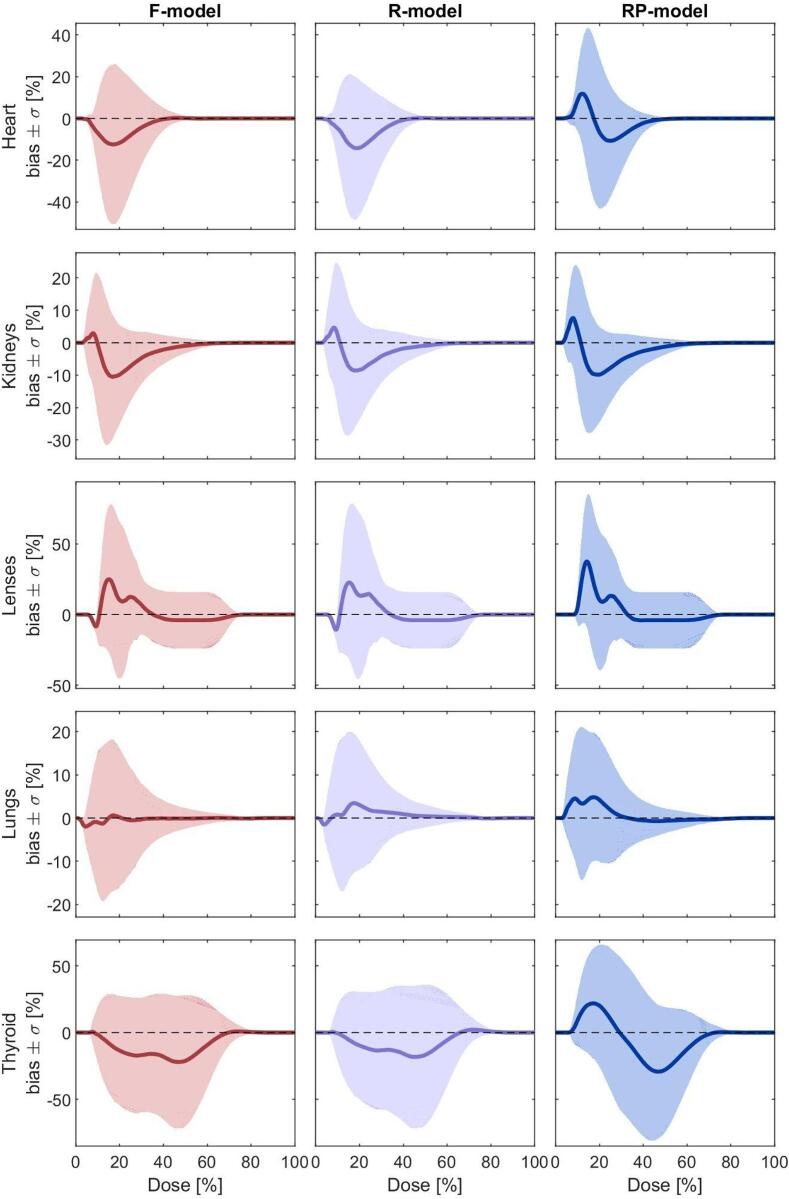
Models’ performance comparison on the validation set. The thick line represents the estimated bias (mean prediction deviation) while the shaded area represents the sigma (variance of the prediction deviation).

Bias and standard deviation (σ) of DVH predictions are shown in Fig. 5. The *F-* and *R-models* had similar bias and σ, with a slight underprediction for the heart, thyroid, and kidneys, and an overprediction for the lenses. The *RP-model* showed comparable trends but slightly increased σ for some OARs. Above 70 % of the prescription dose, all models achieved near-zero bias for all OARs.

Low-dose regions (<50 % prescription dose) posed challenges for all models, with substantial variability in prediction success rates across OARs. The *F-model* generally outperformed the *RP-model*, achieving higher prediction success rates for most OARs due to broader prediction bands.

Overall, the *RP-model*’s precision makes it suitable for standardizing planning practices, while the *F-model*’s ability to encompass clinical variability makes it better suited for RTQA and multicentric efforts.

## 4 Discussion

CSI planning is complex due to the need to balance adequate target coverage with the protection of numerous OARs. This study aimed to develop and evaluate robust KBP models tailored for CSI to standardize planning practices. Challenges stemmed from the wide variability in anatomical volume ratio between the target and the OARs across the pediatric age spectrum (see Supplementary Fig. 2) and the inherent difficulties of multicentric studies, including variations in clinical practices, treatment techniques, and the extended data collection period [5], [13], [14], [19], [9], [10], [11], [26], [27], [28].

Patient size variability dominated other sources of variation, making it difficult to disentangle the effects of clinical practices and techniques. This dominance also constrained statistical comparisons in size-homogeneous subsamples, which lacked significance due to the limited sample size.

To address these challenges, we developed three KBP models: the *F-model* trained on the full dataset, the *R-model* trained on a reduced dataset, and the *RP-model* trained on KBP-guided replanned cases. These models were designed to explore the effects of sample size and standardization on model quality and performance.

Reducing the sample size from the *F-model* to the *R-model* only marginally affected model quality. Both models demonstrated similar R^2^, χ^2^, and MSE values, as well as comparable prediction bias and standard deviation on the validation set (Supplementary Table 2 and Fig. 5). This suggests that reducing the dataset by one-third preserved the anatomical variability essential for training robust models. However, neither model proved particularly precise, as broader variability in clinical practices limited their predictive precision.

The *RP-model*, developed through replanning based on KBP-driven optimization, significantly improved precision and overall quality. It achieved the highest R^2^ and lowest MSE values (Fig. 3) due to the reduced variability in its training library. However, its precision came at the cost of reduced accuracy, as evidenced by narrower prediction bands (Fig. 4) and lower prediction success rates on the validation set (Fig. 5 and Supplementary Fig. 3). These trade-offs indicate that while the *RP-model* is ideal for harmonizing practices and ensuring consistency across centers, it may not adequately capture broader clinical variability [4], [7], [25], [26]. We can conclude that, while model precision can be improved by enlarging the sample size [22], [29], [30], [31], model accuracy can only be improved by carefully reducing the dosimetric variability of the sample [11], [32], [33], [34], [35], [36].

The choice of model depends on its intended application. Models like the *RP-model*, with its homogeneity and narrower prediction bands, are suited for introducing new practices or supporting centers with limited experience, as it reduces inter-planner variability and promotes consistency. Conversely, models like the *F-model*, which encompasses the full variability of clinical data, are more appropriate for RTQA programs and multicentric clinical trials where variability must be accounted for [7], [8], [37], [38], [39].

The intrinsic anatomical variability in CSI, driven by patient-specific differences and the wide age range, complicates defining constraints for treatment. A key contribution of this work is the use of shared KBP models as benchmarking tools to assess treatment feasibility and improve plan quality across clinical environments, including hospitals with low CSI patient volumes [40]. The replanning process further demonstrated that KBP-driven automated planning provides a reliable starting point for CSI plans, ensuring robust, consistent, and high-quality outcomes [18], [41], [42].

To validate these findings, testing the models on external cohorts, including clinical trial participants such as those in the QUARTET project, is crucial to confirm their robustness and generalizability. Expanding KBP models for quality assurance could streamline plan review processes and optimize workflows, reducing the need for recalculations or re-optimizations while improving efficiency.

This work underscores the potential of KBP models as versatile tools for radiotherapy optimization and overall treatments quality improvement. By addressing specific needs, these models facilitate equitable cancer care and improved treatment outcomes. Federated learning approaches and the standardization of clinical procedures are recommended to improve model accuracy and precision further [43], [44]. To support these efforts, the KBP models developed in this study are freely available in Zenodo repository providing a shared resource for the radiotherapy community [23].

## Declaration of Generative AI and AI-assisted technologies in the writing process

During the preparation of this work the author(s) used ChatGPT-4o and DeepL Write in order to check the spelling and improve the legibility of the text. After using this tool/service, the author(s) reviewed and edited the content as needed and take(s) full responsibility for the content of the publication.

## CRediT authorship contribution statement

**Paolo Caricato:** Methodology, Formal analysis, Investigation, Data curation, Writing – original draft, Visualization. **Francesca Cavagnetto:** Conceptualization, Methodology, Investigation, Writing – original draft. **Silvia Meroni:** Conceptualization, Methodology, Investigation, Writing – original draft. **Salvina Barra:** Validation, Resources. **Laura Brambilla:** Investigation, Resources, Writing – review & editing. **Enrica Bovo:** Validation, Resources. **Samuele Cavinato:** Software, Formal analysis, Investigation, Data curation, Writing – original draft. **Alessio Cirone:** Formal analysis, Investigation, Writing – review & editing. **Flavio Giannelli:** Validation, Resources. **Marta Paiusco:** Conceptualization, Writing – review & editing, Supervision, Funding acquisition. **Emilia Pecori:** Validation, Resources. **Emanuele Pignoli:** Conceptualization, Writing – review & editing, Supervision. **Margherita Pollara:** Investigation, Resources, Writing – review & editing. **Giovanni Scarzello:** Validation, Resources. **Alessandro Scaggion:** Conceptualization, Methodology, Formal analysis, Data curation, Writing – original draft, Writing – review & editing, Visualization, Project administration, Funding acquisition.

## Declaration of competing interest

The authors declare that they have no known competing financial interests or personal relationships that could have appeared to influence the work reported in this paper.
