# Supplementary material for: Critical assessment of knowledge-based models for craniospinal irradiation of paediatric patients
Source: Phys Imaging Radiat Oncol. 2025 Jan 20;33:100703. doi: 10.1016/j.phro.2025.100703 (PMC11804776; doi:10.1016/j.phro.2025.100703)

**Supplementary material for *Critical assessment of KBP models for QA of CSI irradiation of pediatric patients***

**A. Details of clinical historical treatments**

**Patients sample**

***Supplementary Table 1****. Detail of patient sample composition.*

| **Center** | **# Patients** | **M/F** | **Age distribution [years]** | | **Planning Technique** | |
| --- | --- | --- | --- | --- | --- | --- |
|  |  |  | **Avg ± st.dev** | **[min;max]** | **VMAT** | **HT** |
| Inst1 | 37 | 26 / 11 | 9.6 ± 4.5 | [3.2 ; 21.0] | 0 | 37 |
| Inst2 | 39 | 29 / 10 | 15.0 ± 10.5 | [2.7 ; 48.3] | 39 | 0 |
| Inst3 | 37 | 16 / 21 | 13. 5± 12.2 | [3.2 ; 21.0] | 19 | 18 |

Included patients suffered from the following pathologies: Medulloblastoma, Germinoma, Ependymoma, Astrocytoma, and Rhabdomyosarcoma, Xantoastrocitoma.

The usual clinical practices employed for CSI treatment at the three centers involved in this study are described hereafter. Each institute's unique treatment planning geometry, Treatment Planning System (TPS), dose algorithms, dose grid size, energy settings, Multi-Leaf Collimator (MLC) configuration, beam gantry angles, and numbers and locations of isocenters are outlined in detail.

**Inst1 - Set-up and planning practices**

At Institute 1, treatment planning is performed with HT techniques. Optimization is performed with Precision TPS (v.3.3.1.3), treatment is delivered using the Radixact and HiArt systems (Accuray, Sunnyvale, CA, USA) with a 6-FFF MV beam and a nominal dose rate respectively of 1180 and 850 cGy/min. A single-isocenter geometry is employed, with sometimes an entry structure to avoid patient arms positioned along the hips (depending on patient immobilization). Dose calculation utilizes the Convolution Superimposition dose algorithm, with a dose grid size of 0.25 cm. The planning parameters include a field width of 2.5 or 5 cm, depending on the patient height, pitch selection is guided by established protocols, and the modulation factor is usually held around 2.

**Inst2 - Set-up and planning practices**

At Institute 2 CSI treatments are performed with VMAT technique using Eclipse TPS (v.15.6, Varian Medical Systems, Inc., Palo Alto, CA). Patients are positioned on individualized foam cushions. The head is immobilized using a carbon fiber headrest and thermoplastic mask. Wedge-shaped knee cushions are used to improve pelvis alignment.

Concerning planning geometry, two or three isocenters are used depending on the length of the target. Only longitudinal shifts are applied between the isocenters. The first isocenter is set in the middle of the brain, the remaining isocenters are placed at about one-third and two-thirds of the spine length respectively. Two arcs are used for each isocenter, one clockwise (CW) and one counter-clockwise (CCW). Collimator angle is set to 90˚ for brain arcs, 2°/358˚ for upper spine arcs, and 4˚/356˚ for lower spine arcs to facilitate arc overlapping and limit the “tongue and groove” effect. Avoidance sectors are used to avoid direct irradiation of the arms. The overlapping region of adjacent fields is about 2.5 cm or more at the isocenter level. A junction shift of a few centimeters is performed after 7-8 fractions. The treatment plans are optimized to achieve PTVs dosimetric objectives, while OARs doses are minimized without compromising target coverage. Each treatment phase is optimized to individually satisfy dosimetric objectives.

**Inst3 - Set-up and planning practices**
At Institute 3, treatment planning is performed with both HT and VMAT techniques. These were optimized in RayStation (v. 9.1 or 10.1, RaySearch Medical Laboratories, Stockholm, Sweden) and Eclipse (v. 15.5 or 16.1, Varian Medical Systems, Inc., Palo Alto, CA) TPS, respectively.

For HT plans, treatment is delivered using the Radixact system (Accuray, Sunnyvale, CA, USA) with a 6-FFF MV beam and a nominal dose rate of 850 cGy/min. A single-isocenter geometry is employed, with a "no-entry" structure to avoid patient arms positioned along the hips. Dose calculation utilizes the Collapsed Cone dose algorithm version 5.1, with a dose grid size of 0.25 cm. The planning parameters include a field width of 2.5 or 5 cm, depending on the patient height, pitch selection is guided by established protocols, and the modulation factor is usually held around 2 to grant a total delivery not larger than 12 minutes.

For VMAT plans, treatments are delivered using a UNIQUE or TrueBeam STX platform (Varian Medical Systems, Inc., Palo Alto, CA) with a 6 MV beam and a nominal dose rate of 600 cGy/min, equipped with a Millennium / Millennium HD MLC. The geometry consists of one isocenter for the PTV brain and cranial portion of the PTV spine, and one, two, or three isocenters for the remaining portion of the PTV spine, based on its extension and the available MLC. Two complete arcs are employed for each spinal isocenter while three or four are used for the cranial section. To ensure the dose distribution robustness against relative isocenter shifts, the plan geometry is conceived to ensure field overlap larger than 5 cm field to obtain smooth dose gradients facilitated by Eclipse's auto-feathering function. To mitigate the presence of patient arms positioned along the hips, the avoidance sector function is used for all the spinal arcs. Dose calculation utilises the Acuros XB algorithm with a dose grid size of 0.25 cm.

**B. Standard plan geometry**

The model training was conducted using selected treatment plans exported from the original TPS and linked to a virtual VMAT plan within Eclipse (v.16.1). The standard geometry employed in this study consists of a first isocenter at the skull base, referenced by four full coplanar arcs. Two arcs, with collimator angles of 87/93 degrees, encompass the entire brain region, and two additional arcs, with collimator angles of 357/3 degrees, target the upper segment of the PTV spine and part of the PTV brain. For each remaining segment of the PTV spine, two full coplanar arcs with complementary collimator angles of 357/3 degrees were incorporated. The number of arcs was dependent on the length of the target, with one or two junctions integrated into the plan to ensure seamless coverage. To maintain consistency, during model building and training all arcs were used without avoidance sectors. A representative illustration of the geometry is given in Supp Fig1.

*
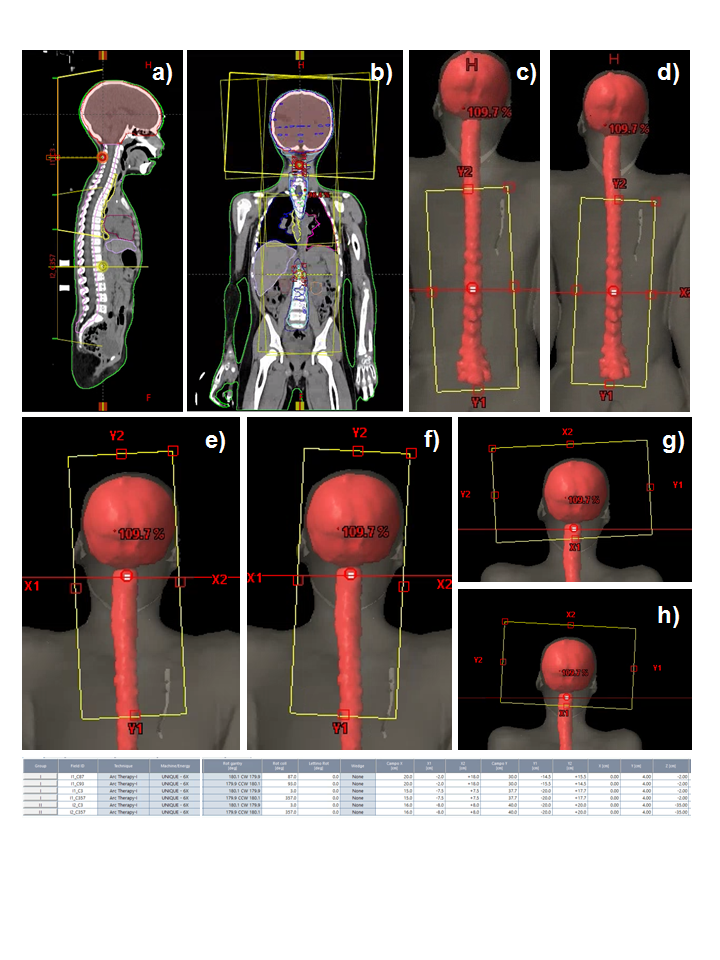
*

***Supplementary Figure1.*** Example of standard plan geometry used to populate the KBP libraries. a) Lateral view. b) Frontal view. c) Spinal arc 1. d) Spinal arc 2. e) Cranial arc 3. f) Cranial arc 4. g) Cranial arc 1. h) Cranial arc 2. Bottom: details of arcs arrangement.

**C. Planning protocol**

The planning protocol here reported is inspired to the SIOP High-Risk Medulloblastoma (SIOP-HRMB) protocol in which the CSI irradiation is scheduled to deliver 39 Gy/30 fx or 36Gy/20 fx. These planning objectives represent an indication of the clinical practice rather than a strict guideline to follow since it is adapted to all the fractionation schemes and the OARs goal are quite conservative with respect to what can be achieved with VMAT and Helical Tomotherapy.

***Supplementary Table 2****. List of Indicative planning objectives.*

| **Structure** | **Goals** |
| --- | --- |
| PTV (Brain and Spine) | V95% ≥ 95% (≥ 90%) |
|  | V90% ≥ 100% |
|  | V107% ≤ 5% (< 10%) |
| Cochlea_L/R | D2% < 35 Gy (at least for one) |
| Kidney_L/R | D2% < 20 Gy |
|  | Dmean < 10 Gy |
|  | V18 Gy < 32% |
| Lens_L/R | D2% < 10Gy (not limiting ) |
| Liver | D2% < 21 Gy |
|  | V6 Gy < 50% |
|  | V21Gy < 20% |
| Lungs | V7 Gy < 50% |
|  | V18 Gy < 25% |
|  | V27 Gy < 22% |
| Parotid_L/R | V26 Gy < 50% |
| Testis_L/R | D2% < 0.5 Gy |
| Breast_L/R | ALARA |
| Glnd_Thyroid | ALARA |
| Heart | ALARA |
| Ovary L/R | ALARA |

**D. Volume distribution**

***Supplementary Figure 2.*** Volume of relevant structures as a function of patient age. Colours mark different centres. Body volume is computed as the volume from the top of the head to the pelvis excluding the arms, percentages are given as a fraction of Body volume. Gray shaded area highlights the non-paediatric age range, i.e. age > 18 years.
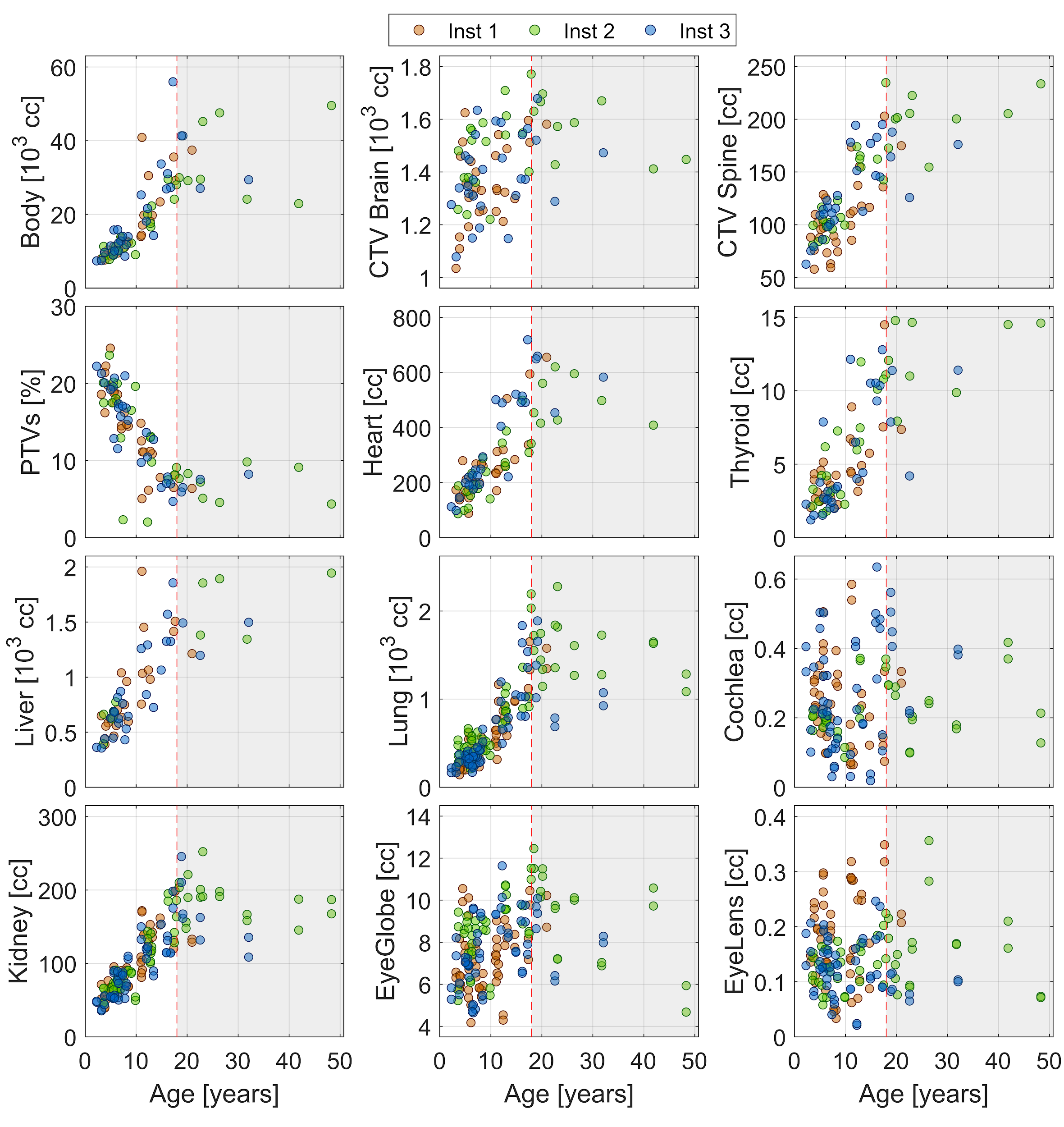


**E. Results of replanning procedure**
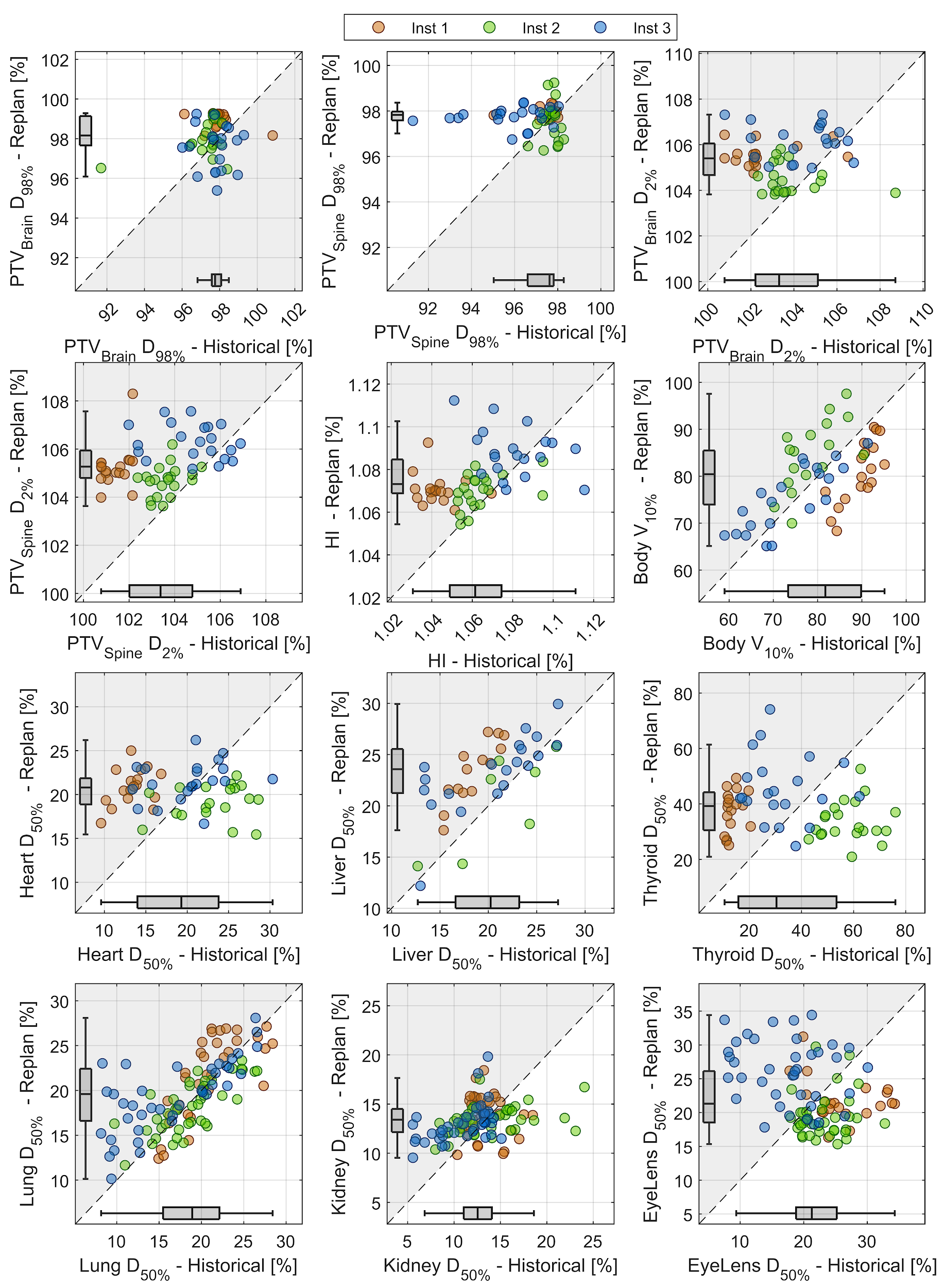


***Supplementary Figure 3****.* Results of the replanning procedure for the main dosimetric endpoints. Historical versus replanned values. Gray shaded area represents a worsening of plan quality. Generally, the dispersion along the Y axis is smaller than the one along the X axis. Bars and whiskers represent the entire population distribution of the original set of plans (along the X axis) and the replanned set of plans (along the Y axis).

**F. Model comparison**

***Supplementary Table 3****.* Relevant characteristics of the libraries of the three trained KBP models. Values are given as mean ± standard deviation and [min;max].

|  | **Full Model** | **Reduced Model** | **Replan Model** |
| --- | --- | --- | --- |
| Gender [M/F] | 32 \ 54 | 26 \ 34 | 26 \ 34 |
| Center [Inst1 / Inst 2 / Inst 3] | 29 \ 29 \ 28 | 18 \ 21 \ 21 | 18 \ 21 \ 21 |
| Age [years] | 11.78±8.33 [2.25;48.30] | 12.09±8.20 [2.26;48.30] | 12.09±8.20 [2.26;48.30] |
| CTV_Brain [cc] | 1420±161 [1030;1770] | 1420±166 [1030;1770] | 1420±165 [1030;1770] |
| CTV_Spine [cc] | 129±44.5 [57.8;235] | 131±46.2 [57.8;235] | 131±46.2 [57.8;235] |
| PTV_Brain [cc] | 1700±182 [1220;2130] | 1710±195 [1220;2130] | 1710±195 [1220;2130] |
| PTV_Spine [cc] | 341±107 [133;622] | 347±113 [133;622] | 347±114 [133;622] |
| EvalBody [10^3 cc] | 19.1±11.4 [7.36;56] | 20±12 [7.36;560] | 19.4±11.3 [7.36;56] |
| Esophagus [cc] | 11.5±6.38 [4.43;29.3] | 12.3±7 [4.43;29.3] | 12.7±7.1 [4.43;29.3] |
| Heart [cc] | 305±162 [86.9;719] | 314±166 [86.9;719] | 318±169 [86.9;719] |
| Liver [cc] | 923±445 [356;1960] | 956±462 [356;1960] | 931±439 [356;1940] |
| Thyroid [cc] | 5.87±3.87 [1.2;14.8] | 6.24±3.94 [1.2;14.8] | 6.2±3.97 [1.2;14.8] |
| Cochlea [cc] | 0.247±0.132 [0.0187;0.635] | 0.244±0.126 [0.0187;0.562] | 0.243±0.125 [0.0187;0.562] |
| EyeGlobe [cc] | 7.86±1.78 [4.18;12.5] | 8.02±1.53 [4.68;11.6] | 8±1.53 [4.68;11.6] |
| EyeLens [cc] | 0.15±0.068 [0.021;0.36] | 0.15±0.063 [0.021;0.35] | 0.14±0.060 [0.021;0.350] |
| Kidney [cc] | 107±49.6 [35.5;252] | 109±48.2 [35.5;252] | 109±48.3 [35.5;252] |
| Lung [cc] | 726±497 [141;2280] | 736±483 [141;2280] | 740±482 [141;2280] |
| Parotid [cc] | 11.2±6.16 [1.2;24.8] | 12.7±6.28 [3.37;24.8] | 12.8±6.37 [3.37;24.8] |

***Supplementary Table 2.*** Comparison of models’ characteristics for each trained OAR.

| **Structure** | **Quantity** | **F-model** | **R-model** | **RP-model** |
| --- | --- | --- | --- | --- |
| Breast (L+R) | Structures (outliers) | 34 (10) | 28 (10) * | 28 (10) * |
|  | Goodness of fit - R2 | 0.456 | 0.459 | 0.583 |
|  | Goodness of fit - X2 | 1.099 | 1.209 | 1.087 |
|  | Goodness of estimation -MSE | 0.0172 | 0.0195 | 0.0067 |
| Cochlea (L+R) | Structures (outliers) | 143 (0) | 85 (1) | 89 (2) |
|  | Goodness of fit - R2 | 0.341 | 0.44 | 0.656 |
|  | Goodness of fit - X2 | 1.043 | 1.024 | 1.087 |
|  | Goodness of estimation -MSE | 0.0058 | 0.0054 | 0.004 |
| Esophagus | Structures (outliers) | 49 (1) | 33 (2) * | 31 (1) * |
|  | Goodness of fit - R2 | 0.264 | 0.449 | 0.607 |
|  | Goodness of fit - X2 | 1.056 | 1.146 | 1.144 |
|  | Goodness of estimation -MSE | 0.0089 | 0.0084 | 0.0024 |
| EyeGLobe (L+R) | Structures (outliers) | 172 (4) | 118 (3) | 117 (2) |
|  | Goodness of fit - R2 | 0.304 | 0.26 | 0.289 |
|  | Goodness of fit - X2 | 1.018 | 1.044 | 1.055 |
|  | Goodness of estimation -MSE | 0.0076 | 0.0096 | 0.0043 |
| EyeLens (L+R) | Structures (outliers) | 163 (1) | 112 (1) | 106 (2) |
|  | Goodness of fit - R2 | 0.25 | 0.26 | 0.191 |
|  | Goodness of fit - X2 | 1.031 | 1.046 | 1.02 |
|  | Goodness of estimation -MSE | 0.0181 | 0.0188 | 0.0163 |
| Heart | Structures (outliers) | 84 (3) | 58 (1) | 59 (1) |
|  | Goodness of fit - R2 | 0.318 | 0.281 | 0.376 |
|  | Goodness of fit - X2 | 1.031 | 1.046 | 1.092 |
|  | Goodness of estimation -MSE | 0.0073 | 0.0068 | 0.0017 |
| Kidney (L+R) | Structures (outliers) | 172 (2) | 119 (0) | 118 (5) |
|  | Goodness of fit - R2 | 0.268 | 0.234 | 0.536 |
|  | Goodness of fit - X2 | 1.02 | 1.028 | 1.043 |
|  | Goodness of estimation -MSE | 0.0019 | 0.0025 | 0.0009 |
| Liver | Structures (outliers) | 64 (0) | 42 (0) | 41 (2) |
|  | Goodness of fit - R2 | 0.479 | 0.426 | 0.679 |
|  | Goodness of fit - X2 | 1.06 | 1.063 | 1.085 |
|  | Goodness of estimation -MSE | 0.0014 | 0.0017 | 0.0017 |
| Lung (L+R) | Structures (outliers) | 173 (3) | 119 (3) | 119 (5) |
|  | Goodness of fit - R2 | 0.439 | 0.422 | 0.48 |
|  | Goodness of fit - X2 | 1.024 | 1.034 | 1.034 |
|  | Goodness of estimation -MSE | 0.0022 | 0.0022 | 0.0014 |
| Parotid (L+R) | Structures (outliers) | 63 (2) | 46 (1) | 50 (1) |
|  | Goodness of fit - R2 | 0.493 | 0.3 | 0.547 |
|  | Goodness of fit - X2 | 1.055 | 1.065 | 1.089 |
|  | Goodness of estimation -MSE | 0.0064 | 0.0064 | 0.0031 |
| Thyroid | Structures (outliers) | 86 (2) | 60 (2) | 60 (1) |
|  | Goodness of fit - R2 | 0.155 | 0.11 | 0.243 |
|  | Goodness of fit - X2 | 1.034 | 1.023 | 1.023 |
|  | Goodness of estimation -MSE | 0.0394 | 0.0453 | 0.0129 |

***Supplementary Figure 4.*** Model’s performance comparison on the validation set. The thick line represents the point-by-point prediction success rate. 68% (standard deviation) and 50% (Interquartile range) lines are reported as reference.
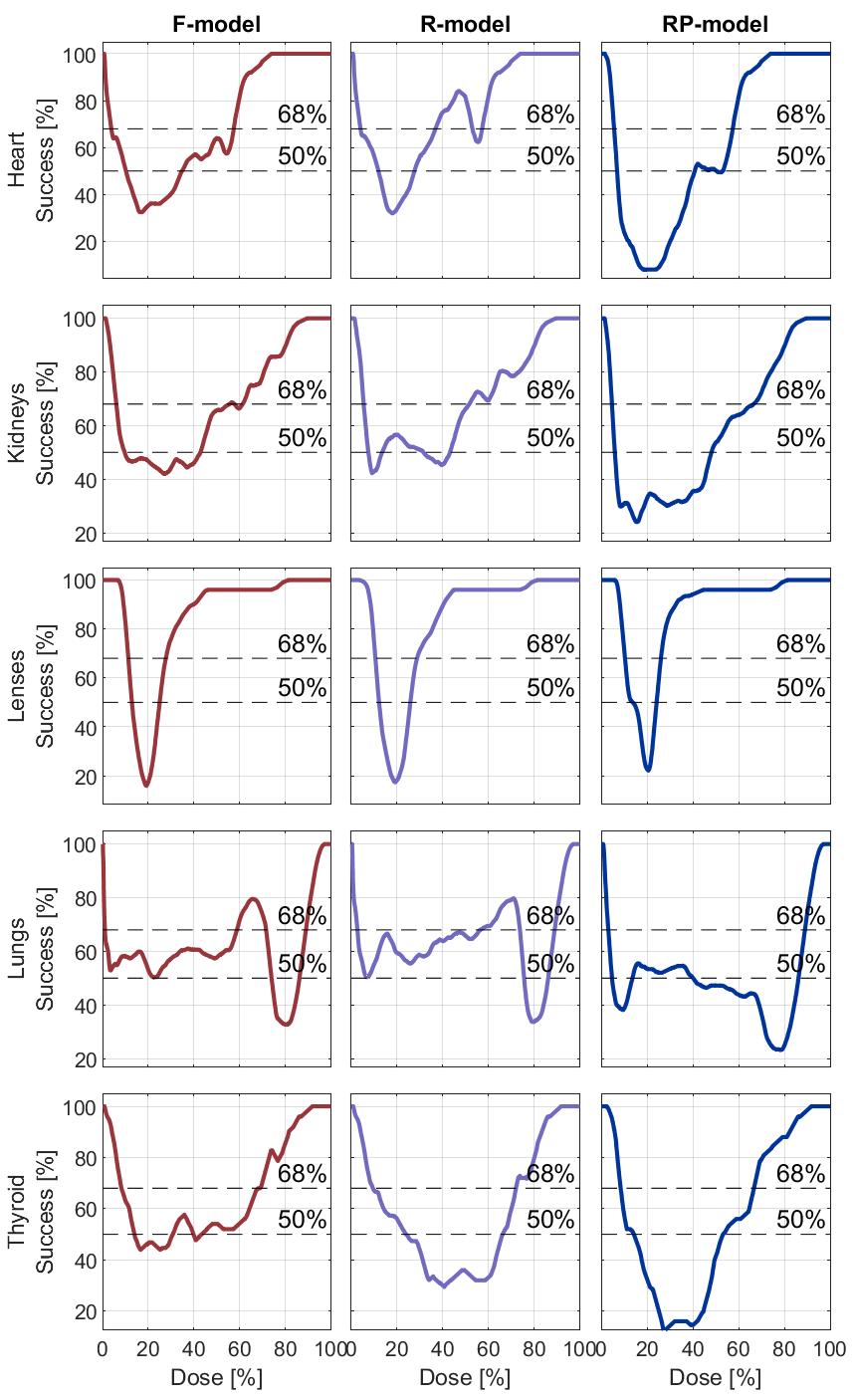

Supplement: Supplementary Data 1 [file mmc1.docx]
